# Estimation of maize evapotraspiration under drought stress - A case study of Huaibei Plain, China

**DOI:** 10.1371/journal.pone.0223756

**Published:** 2019-11-05

**Authors:** Hongwei Yuan, Yi Cui, Shaowei Ning, Shangming Jiang, Xianjiang Yuan, Guangmin Tang

**Affiliations:** 1 Key Laboratory of Water Conservancy and Water Resources of Anhui Province, Water Resources Research Institute of Anhui Province and Huaihe River Commission, Ministry of Water Resources, Hefei, China; 2 Stage Key Laboratory of Hydraulic Engineering Simulation and Safety, Tianjin University, Tianjin, China; 3 School of Civil Engineering, Hefei University of Technology, Hefei, China; 4 Interdisciplinary Centre for River Basin Environment, University of Yamanashi, Kofu, Japan; Harran University, TURKEY

## Abstract

Given the importance and complexity of crop evapotranspiration estimation under drought stress, an experiment tailored for maize under drought stress was completed using six sets of large-scale weighing lysimeters at the Xinmaqiao Comprehensive Experimental Irrigation and Drainage Station, Anhui Province, China. Our aim was to analyze maize evapotranspiration under different drought conditions. Based on estimates of maize evapotranspiration under no drought stress using the dual crop coefficient approach, we optimized and calibrated basic crop coefficients *K*_*cb*ini_, *K*_*cb*mid_, *K*_*cb*end_, and the maximum crop coefficient *K*_*c*max_ using a genetic algorithm. Measurements of solar radiation at the experimental station were used to derive the empirical parameters *a* and *b* from the Angstrom formula through the genetic algorithm, and then evapotranspiration was calculated for the reference crop (ET_0_). We then estimated the maize evapotranspiration under drought using the dual crop coefficient approach. The results indicated that a slight water deficit during the earlier stage of vegetative growth may stimulate the maize homeostatic mechanism and increase tolerance to drought stress in later growth periods. Maize evapotranspiration significantly decreased if drought stress continued into the elongation stage, and the same degree of drought stress had a greater influence on the middle and later stages of vegetative and reproductive growth. The calibrated results for *K*_*cb*ini_, *K*_*cb*mid_, *K*_*cb*end_, and *K*_*c*max_ were 0.155, 1.218, 0.420 and 1.497 respectively. We calculated the root-mean-square error (RMSE), mean absolute error (MAE), and mean relative error (MRE) of maize evapotranspiration under no drought stress over the full growing season using a dual crop coefficient approach, and the results were 1.33 mm/day, 0.99 mm/day, and 1.30%, respectively, or 18.40%, 17.50%, and 91.11% lower than results using the recommended coefficients. The RMSE, MAE, and MRE results for maize under drought stress during two full growth periods were 1.18 mm/day, 0.98 mm/day, and 13.92%, respectively. These results were higher than maize without drought stress, but better than the estimated results based on FAO-56 recommended values. Therefore, maize evapotranspiration estimation under drought stress using the dual crop coefficient approach and genetic algorithm was reasonable and reliable. This study provides a theoretical basis for developing suitable regional irrigation programs and decreasing losses due to agricultural drought.

## Introduction

Summer maize is one of the main food crops and the most important forage crop in China’s Huaihe River Basin. The summer maize growth period runs from June to September when average temperatures and evapotranspiration rates are high. Water stress occurs easily in the soil during years with poor rainfall. The Huaihe River Basin is a transitional zone of northern and southern climates, with high and low latitudes facing both the ocean and inland. Influenced by monsoons and landforms, the spatial and temporal distributions of rainfall in this area are extremely uneven[1,2]. The specific climatic conditions, geographical environment, basin characteristics, and influence of human activities have resulted in frequent droughts along the Huaihe River Basin throughout history, posing a severe threat to food production security and social stability [3,4]. Especially since the 1990s, droughts have become increasingly frequent, and with economic and societal development the losses caused by drought are more serious. During the 62 years from 1949 to 2010, the accumulated drought-stricken area in the whole basin was 167 million ha, the disaster-affected area was 87.30 million ha, and total crop losses were 1.396 billion kg. On average, 2.698 million ha of crops suffered from drought and 1.408 million ha of crops were affected by disaster, resulting in widespread reductions in output, seasonal crop failures, and even total crop failures [5–9]. Drought has become a restriction to sustainable development of the agricultural economy in the basin. For this reason, understanding and estimating maize evapotranspiration under drought stress can improve the development of irrigation programs, increase water use efficiency, and guarantee high and stable yields of maize in the Huaibei Plain [10–12].

Estimating crop evapotranspiration under drought stress has been a long-term research focus in farmland irrigation and has received attention globally [11,13–15]. Computational methods mainly include aerodynamic methods, the Bowen ratio-energy balance method, and remote sensing. The crop coefficient approach recommended by the Food and Agriculture Organization (FAO) has extensive applicability [16–21]. As an empirical method, the dual crop coefficient approach is characterized by easy operation, reliable precision, and practicability. Able to separate crop transpiration from soil evaporation, this approach has been adopted worldwide [22–25]. However, the dual crop coefficient approach is mainly used to estimate crop evapotranspiration for crops that are not under water stress, and few studies have been conducted on food crops under drought stress [26, 27]. Although the dual crop coefficient approach may be used to adjust the recommended FAO values according to local environmental and climatic conditions, there is a difference between the estimated and measured evapotranspiration values [28,29]. The computational method used to determine reference crop evapotranspiration (ET_0_) usually adopts the Penman formula, which involves the computation of solar radiation. The parameters *a* and *b* in the formula use FAO-56 recommended values, which are not necessarily suitable for all areas; therefore, their calculation should be based on the actual measurement of solar radiation [30,31]. A genetic algorithm (GA) is a search algorithm that simulates the genetic and evolutionary processes of the biological world. GAs apply the “survival competition and survival of the fittest” competition mechanism [32–34] and are highly parallel, random, and adaptive. A GA can automatically acquire knowledge about the search space during the search process, adaptively obtain an optimal solution, and is not limited by the model structure, constraints, initial values, number of parameters, or the target function value. Auxiliary continuous, steerable, and single peak information is especially suitable for managing complex functions and combinatorial optimization. As a product of multidisciplinary integration, GAs are a self-organizing, adaptive, and comprehensive analytical technique that are widely used in agricultural water management. At present, GAs have been widely applied for water resources allocation and irrigation system optimization in irrigation districts, but related research on crop evapotranspiration is scarce [35–47].

Six sets of large-scale weighing lysimeters at the Xinmaqiao Comprehensive Experimental Station of Irrigation and Drainage were used to create different combinations of drought test programs, conduct a special irrigation experiment for maize under drought stress, and analyze maize evapotranspiration under different drought conditions. After estimating maize evapotranspiration under no drought stress using the dual crop coefficient approach, we employed a GA to derive crop coefficients and validated our method using the estimated results for maize evapotranspiration under drought stress. We aimed to investigate the response of maize evapotranspiration under continuous and combined drought stress, and the adaptive compensation mechanism after water returned to generate a method for estimating crop evapotranspiration under drought stress. The goal is to provide a theoretical basis for developing regional irrigation programs and decreasing losses due to agricultural drought.

## Materials and methods

### Overview of experimental site

**Fig 1:**
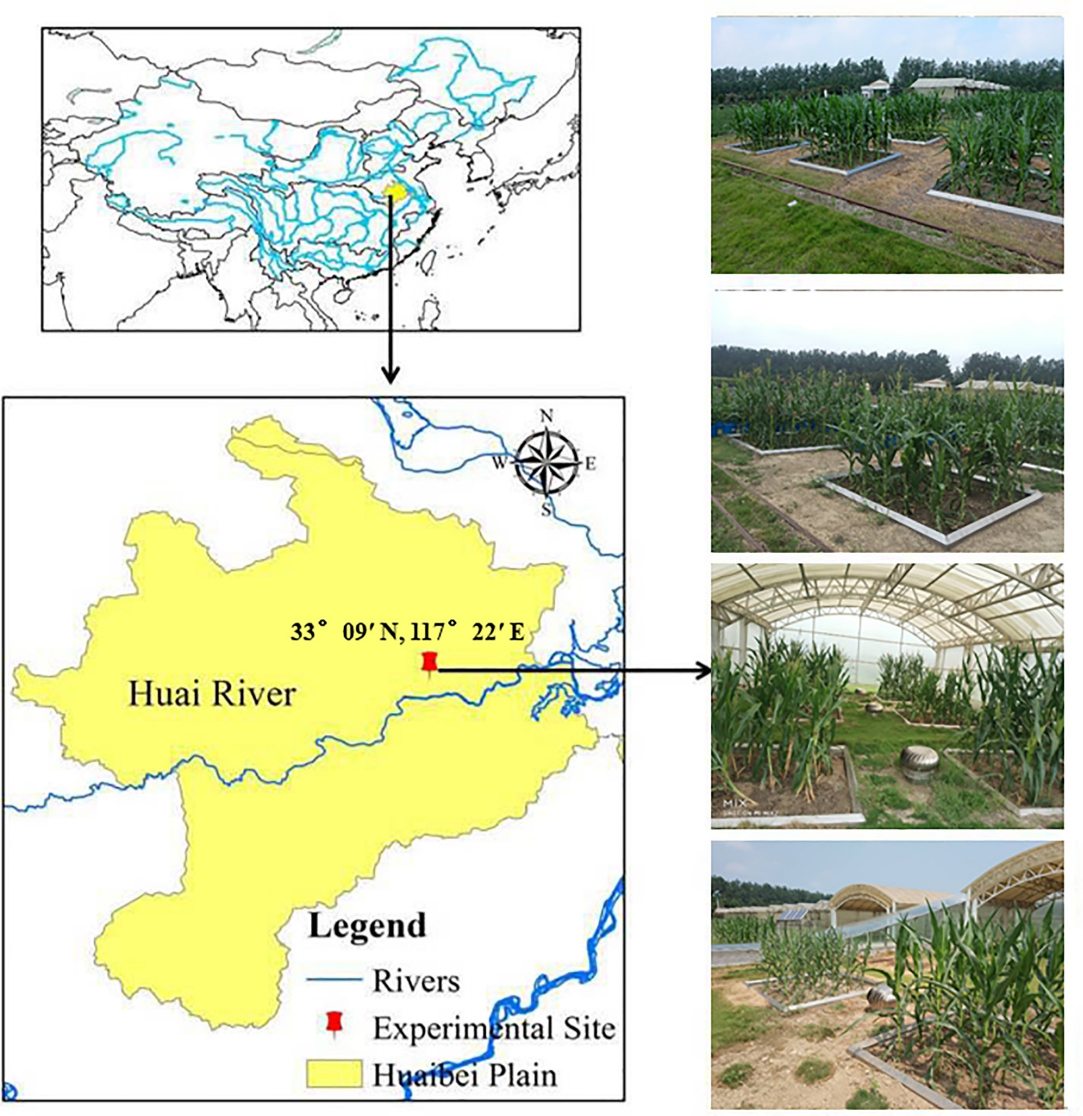
Location of the experimental site in the Huaibei Plain and conditions of the maize plots.

The experiment was carried out at the Xinmaqiao Comprehensive Experimental Station of Irrigation and Drainage, Institute of Hydraulic Research of the Huaihe River Commission, which operates under China’s Ministry of Water Resources, from June to September 2017. The station is located in the south central part of the Huaibei plain, with an altitude of 19.7 m (33°09′ N, 117°22′ E)(Fig 1). It is a semi-arid and semi-humid monsoon climatic region. The mean annual precipitation is 917 mm and precipitation from June to September accounts for about 60–70% of the annual total precipitation. Annual evaporation is 916 mm, groundwater is found at a depth of 1.0–3.0 m, and the mean annual temperature is 15.0°C. The soil in the experimental area is typical lime concretion black soil of the Huaibei plain. The surface layer soil has a depth of 0–20 cm and is made up of 6.9% sand, 52.8% soil, and 40.3% clay. The soil bulk density is 1.36 g/cm^3^, field capacity is 38.1% (percentage of volume water content), and water content at the wilting point is 16.6% (percentage of volume water content). In addition, the thick and heavy texture, poor structure, solid soil body, and fracture development cause the soil to have poor water retention capacity and dry easily.

### Experiment design

Maize evapotranspiration tests under drought stress were carried out using six sets of large-scale weighing lysimeters at the Xinmaqiao Comprehensive Experimental Station of Irrigation and Drainage. The lysimeters were 2 m long, 2 m wide, and 2.3 m deep, and each had a cover to protect it from rainfall. The soil moisture was under manual irrigation control during the experiment. The maize variety for the experiment was Longping No. 206, which was seeded on June 9, 2017 and harvested on September 17, 2017 for a total growth period of 101 days. Maize growth during the experiment was divided into four stages: seedling (stage I, 9 June to 9 July, 31 days in total), jointing (stage II, 10 July to 27 July, 18 days in total), reproductive growth (stage III, 28 July to 13 August, 17 days in total), and filling and ripening (stage IV, 14 August to 17 September, 35 days in total). The control factor of the experiment was soil moisture content during the growth stage, and different lower limits were set. According to the multi-year drought irrigation experiments at the station, four drought levels categories had been determined: no drought, slight drought, medium drought, and severe drought, with respective lower limits of soil moisture content of 70%, 55%, 45%, and 35%. Here, the soil moisture content lower limits referred to the percentage of soil moisture content relative to field capacity. Experimental details are shown in Table 1. We applied 300 g compound fertilizer and 120 g urea to the area around each lysimeter. The planting density of maize was 20 plants/pit, with four rows for each test pit. To reflect actual irrigation conditions, when the soil moisture content of the experimental plot reached the corresponding lower control limit, we irrigated the farmland until it reached field capacity. All other management methods were identical except, to guarantee normal maize growth and development and protect it from diseases and insect pests.

**Table 1:** Experiment implementation.

Treatment	Soil Moisture Content Lower Limits at Different Growth Stages (%)			
	Stage I	Stage II	Stage III	Stage IV
**T1**	55	55	55	45
**T2**	55	45	45	35
**CK(no drought)**	70	70	70	70

### Collection of experimental data

#### Meteorological data

We used the automatic meteorological station (WS-STD1 manufactured by DELT-T Company, Cambridge, UK), which is 2 m from the ground, to measure meteorological data, including average wind velocity (*u*_2_, m/s), average temperature (*T*,°C), relative humidity (*R*_*h*_, %), and total solar radiation (*R*_*s*_, MJ/(m^2^·d)). We collected data every 5 s and recorded it in a data acquisition unit every 1 h.

#### Soil moisture content

The soil moisture content at a depth of 0–40 cm was measured by manual soil sampling, and the data at depths of 40, 60, and 80 cm were measured using a soil moisture sensor embedded inside the lysimeter. Finally, we derived the mean soil moisture content in a 0–60 cm soil layer. We measured the average soil moisture content once every five to seven days, and increased the time for measurement in growth stages when large amounts of soil moisture were consumed.

#### Evapotranspiration

Actual maize evapotranspiration was measured by the large-scale weighing lysimeters, model QYZS-201 (Xi'an Qing Yuan Company, Xi'an, China). There were six sets in total, the area of each set was 2 m × 2 m = 4 m^2^, the depth was 2.3 m, mass was about 15 t, and measurement accuracy was 0.02 mm. The acquisition system was used to automatically collect and record data at a 1 h time interval, and daily evapotranspiration was obtained by summing the data of 24 h.

#### Irrigation amount

The irrigation amount under treatment *I* (mm) is calculated as:

$$I=1000(\theta_{FC}-\theta_{i})Z_{r}$$ (1)

where *θ*_FC_ is the field capacity of the evaporation layer, m^3^/m^3^; *θ*_*i*_ is the soil moisture content measured before irrigation, m^3^/m^3^; and *Z*_*r*_ is the planned depth of the wet layer, m, set to 0.6 m here.

The irrigation amount was controlled by a water meter at the start of the pipeline.

### Angstrom formula parameter calibration using genetic algorithm

#### Angstrom formula and ET_0_ calculation

The Angstrom formula was developed by Angstrom in 1922, and introduced to China by Dakang et al. [30,31]:

$$R_{s}=(a+bS)R_{a}$$ (2)

where *S* is the sunshine percentage, i.e., the ratio of actual to theoretical hours of sunshine; *R*_a_ is the solar radiation at the edge of the atmosphere, MJ/(m^2^·day); and *a* and *b* are empirical parameters that reflect the attenuation characteristics of radiation travelling from space through the atmosphere.

Solar radiation at the edge of the atmosphere refers to the solar radiation that reaches the upper limit of the atmosphere. Its distribution and change are free from atmospheric influence, and are mainly influenced by earth–sun distance, solar altitude, and day length. This paper used the total amount of daily astronomical solar radiation to represent *R*a.

ET_0_ is calculated by the Penman-Monteith formula:

$$ET_{0}=\frac{0.408\Delta (R_{n}-G)+\gamma \frac{900}{T+273}u_{2}(e_{s}-e_{a})}{\Delta +\gamma (1+0.34u_{2})}$$ (3)

$$R_{n}=(1-\alpha )R_{s}-R_{n1}$$ (4)

where *ET*_0_ is reference crop evapotranspiration, mm/d; *R*_n_ is the crop surface net radiation, MJ/(m^2^·day); *G* is the soil heat flux, MJ/(m^2^·day); *e*_s_ is the saturation water vapor pressure, kPa; *e*_a_ is the actual water vapor pressure, kPa; Δ is the saturation water vapor pressure slope and temperature curve, kPa/°C; *γ* is the psychrometer constant; *α* is the reference crop reflectivity, here set to 0.23; and *R*_*nl*_ is the net long-wave radiation, MJ/(m^2^·day).

For computational equations for the other variables in Eqs (3) and (4) please refer to SL 13–2015 Specifications for Irrigation Experiment [48].

### Parameter calibration method

We selected the least squares method and GA to calibrate parameters. The least square method is a coefficient calibration method generally used in similar studies. Based on the solar radiation at the edge of the atmosphere *R*_*a*_ and the actual *R*_*s*_ and *S*, we obtained values for *a* and *b* using the least squares regression fit Eq (2).

We chose empirical coefficients *a* and *b* as the optimization variables, and the actual value for *R*_*s*_*/R*_*a*_ at Xinmaqiao Experimental Station and sunshine percentage *S* were the objective function. Values for *a* and *b* in the Huaibei plain were solved using GA. The specific procedure is as follows:

$$\min f(a,b)=\sum_{i=1}^{n}\left|X_{i}(a,b)-Y_{i}\right|$$ (5)

$$s.t.\{\begin{array}{c} 0\leq a\leq 1\\ 0\leq b\leq 1 \end{array}$$ (6)

where *X*_*i*_ is the sunshine percentage *S* on the *i*th day, *Y*_*i*_ is the ratio of total solar radiation to the solar radiation at the edge of the atmosphere on the *i*th day (R_*s*_/R_*a*_), and *n* is the total daily solar radiation.

### Estimating maize evapotranspiration using a dual crop coefficient approach and genetic algorithm

We calculated maize evapotranspiration by the dual crop coefficient approach [49]:

$$ET_{c}=(K_{s}K_{cb}+K_{e})ET_{0}$$ (7)

where *ET*_*c*_ is crop evapotranspiration, mm/day; *K*_*s*_ is the soil water stress coefficient, reflecting the influence of soil moisture content in the root zone on crop evapotranspiration, 0 < *K*_*s*_ ≤ 1, when soil moisture content has no influence on crop growth, *K*_*s*_ = 1; *K*_*cb*_ is the basic crop coefficient, which is the ratio of *ET*_*c*_ to *ET*_0_ when the soil surface is dry and the average soil moisture content in the root zone meets transpiration requirements; and *K*_*e*_ is the soil surface evaporation coefficient, which reflects the short term influence of an increase in soil surface evaporation on ET_c_ due to surface soil moisture after irrigation or rainfall.

### Basic crop coefficient determination

The FAO recommends that the total growth period of maize should be divided into four growth stages: initial growth, rapid development, middle growth, and mature. The single-point value of *K*_*cb*_ is then calculated for the initial, middle, and mature stages as *K*_*cb*ini_, *K*_*cb*mid_, and *K*_*cb*end_, respectively. The intermediate value is obtained by linear interpolation [49]. According to related research and combined with the actual growth conditions of maize in this experiment, the lengths of the different growth stages were determined as shown in Table 2. The following maize crop coefficients in different growth stages and under standard conditions are recommended by FAO-56: *K*_*cb*ini_ = 0.15, *K*_*cb*mid_ = 1.15, and *K*_*cb*end_ = 0.50. When *R*_*H*min_ is greater than 45% or the wind velocity exceeds 2 m/s, any *K*_*cb*mid_ and *K*_*cb*end_ greater than 0.45 should be corrected by using the following equation:

$$K_{cb(Adj)}=K_{cb(Tab)}+[0.04(u_{2}-2)-0.004(R_{hmin}-45)]{\left(\frac{h}{3}\right)}^{0.3}$$ (8)

where *K*_*cb(Tab)*_ and *K*_*c*b(Adj)_ are the crop coefficients recommended by FAO-56 that were adjusted according to the climatic conditions at the experimental station, and *h* is the average plant height during growth stages, m.

**Table 2:** Growth stages according to FAO-56 and climatic conditions at the experimental site and plant heights during each stage.

Growth Stage	Initial Stage	Development Stage	Midseason Stage	Late Season Stage
**Number of days in each stage (days)**	16	28	32	25
**Mean wind speed at a height of 2 m, *u***_***2***_ **(m s**^**−1**^**)**	1.136	0.955	0.684	0.740
**Mean minimum relative humidity, RHmin (%)**	52.572	59.880	61.868	62.937
**Mean maize plant height, h (m)**	0.365	1.059	2.082	2.219

#### Soil surface evaporation coefficient

Soil evaporation between plants and within a canopy is controlled by the soil surface energy and potential atmospheric evaporation. The evaporation intensity reaches a peak after rainfall or irrigation. The evaporation intensity of the soil surface decreases quickly as it dries, and *K*_*e*_ is expressed as [49]:

$$K_{e}=\min(K_{r}(K_{c\max}-K_{cb}),f_{ew}K_{c\max})$$ (9)

$$K_{r}=\{\begin{array}{cc} 1 & \left(D_{e,i-1}\leq R_{ew}\right)\\ \frac{T_{ew}-D_{e,i-1}}{T_{ew}-R_{ew}} & \left(D_{e,i-1}>R_{ew}\right) \end{array}$$ (10)

$$T_{ew}=1000(\theta_{FC}-0.5\theta_{WP})Z_{e}$$ (11)

$$R_{ew}=8+0.08C_{l}$$ (12)

$$K_{c\max}=\max\left\{\left\{1.2+\left[0.04\left(u_{2}-2\right)-0.004\left(RH_{\min}-45\right)\right]{\left(\frac{h}{3}\right)}^{0.3}\right\},\left\{K_{cb}+0.05\right\}\right\}$$ (13)

$$f_{ew}=\min(1-f_{c},f_{w})$$ (14)

$$f_{c}={\left[\frac{K_{cb}-K_{c\min}}{K_{c\max}-K_{c\min}}\right]}^{\left(1+0.5h\right)}$$ (15)

where *K*_*r*_—soil evaporation attenuation coefficient [22]; *K*_*c*max_—upper limit crop coefficient after irrigation or rainfall; *f*_*ew*_—ratio of soil area not covered by plant canopy and sufficiently moist after rainfall or irrigation, to the total area; *D*_*e*,*i*-1_—accumulated soil evaporation from the previous day for calculating the days with rainfall or irrigation, mm; *R*_*ew*_—soil evaporation under potential atmospheric evaporation; *T*_*ew*_—maximum water volume of evaporation the soil surface in drought, mm; *Z*_*e*_—soil evaporation layer depth (m), which was combined with the FAO recommended value and the experimental soil conditions, here set to 0.1 m; *θ*_WP_—evaporation layer soil water content at wilting point, m^3^/m^3^; *C*_*l*_—clay particle content in the soil evaporation layer, in the local Shajiang black soil at a depth of 0–10 cm it is 25.42%; *f*_*c*_—maize canopy effective coverage coefficient [23]; and *f*_*w*_—ratio of sufficiently moist surface soil after rainfall or irrigation. We used flood irrigation, and *f*_*w*_ = 1.0 [49]. *K*_*c*min_ is the lower limit of the dry and bare soil crop coefficients, here set to 0.15 [49].

When calculating *K*_*e*_, it is necessary to calculate *D*_*e*,*i*_ [22] according to the daily water volume balance equation for the soil evaporation layer:

$$D_{e,i}=D_{e,i-1}-(P_{i}-R_{oi})-\frac{I_{i}}{f_{w}}+\frac{E_{i}}{f_{ew}}+T_{ew,i}+D_{pe,i}$$ (16)

$$E_{i}=K_{e}ET_{0}$$ (17)

where *P*_*i*_—rainfall on the *i*th day, mm; *R*_*oi*_—rainfall excess on the *i*th day, mm; *I*_*i*_*—*irrigation amount on the *i*th day, mm; *E*_*i*_—average amount of soil evaporation on the *i*th day, mm; *T*_*ew*,*i*_—amount of evaporation obtained from the surface not covered by plants and sufficiently moist on the *i*th day, mm; and *D*_*pe*,*i*_—leakage of surface soil on the *i*th day, mm.

*T*_*ew*,*i*_ can be ignored [49] due to the control of experimental conditions, and *P*_*i*_, *R*_*oi*_, and *D*_*pe*,*i*_ are all equal to 0.

#### Calculating soil water stress coefficient

The soil water stress coefficient is calculated as follows [49]:

$$K_{s}=\{\begin{array}{cc} 1 & \left(D_{r}\leq R_{aw}\right)\\ \frac{T_{aw}-D_{r}}{T_{aw}-R_{aw}} & \left(D_{r}>R_{aw}\right) \end{array}$$ (18)

$$R_{aw}=pT_{aw}$$ (19)

$$T_{aw}=1000(\theta_{FC}-\theta_{WP})Z_{r}$$ (20)

where *D*_*r*_—water volume consumed by the maize root system, mm; *T*_*aw*_—total effective water volume in the root system [49], mm; and *R*_*aw*_—water volume in the root system that is easily absorbed and used, mm, with a value of 0.55 for *p* [49].

When calculating *K*_*s*_, it is necessary to calculate *D*_*r*,*i*_ [46] according to the daily soil water volume balance equation:

$$D_{r,i}=D_{r,i-1}-(P_{i}-R_{oi})-I_{i}-C_{ri}+ET_{c,i}+D_{pi}$$ (21)

where *C*_*ri*_—ascending water in the soil capillary tube on the *i*th day, mm, and *D*_*pi*_—deep seepage on the *i*th day, mm.

Because there was no groundwater recharge in this experiment, *C*_*r*_ = 0. Deep seepage *D*_*p*_ was measured by the lysimeter basement water pipe.

#### Crop coefficient calibration using genetic algorithm

A parameter optimization model was built based on the estimation of maize evapotranspiration under no drought stress (CK) using the dual crop coefficient method. Basic crop coefficients *K*_*cb* ini_, *K*_*cb* mid_, and *K*_*cb* end_, and maximum crop coefficient *K*_*c* max_ were used as optimization variables. The sum of the absolute errors between the daily observed and simulated *ET*_*c*_ in CK over 101 days served as an objective function. These days were randomly selected during each maize growth stage. Then, a GA was applied to solve the function via programing in MATLAB (version R2014a) (MathWorks Company, Natick, USA), and four crop coefficients were obtained. The specific process is shown in Eq (22). Lastly, the crop coefficients were calculated based on actual maize growth at the experimental station.

$$\min\;f(K_{cbini,}K_{cbmid,}K_{cbend,}K_{c\max})=\sum_{j}^{m}\left|X_{j}(K_{cbini,}K_{cbmid,}K_{cbend,}K_{c\max})-Y_{j}\right|$$ (22)

$$s.t.\{\begin{array}{c} 0<K_{cbini}<0.5\\ 1<K_{cbmid}<2\\ 0<K_{cbend}<0.5\\ 1<K_{c\max}<2 \end{array}$$

where *X*_*j*_—estimated maize evapotranspiration under no drought stress (CK treatment) using the dual crop coefficient approach on the *j*th day, mm; *Y*_*j*_—measured maize evapotranspiration measured under no drought stress on the *j*th day, mm; and *m*—length of the total growth stage, 101 days.

## Results and analysis

### Feature analysis of maize evapotranspiration under drought stress

**Fig 2:**
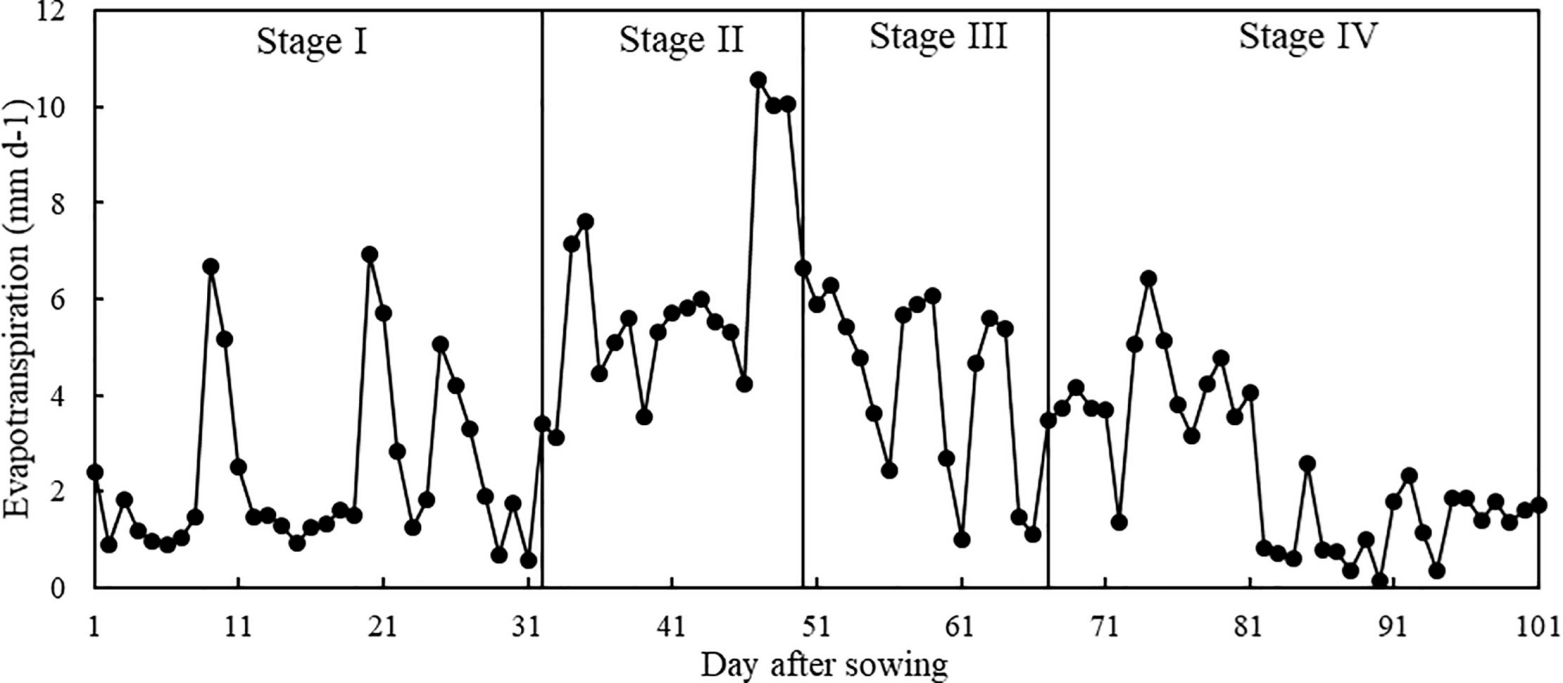
Measured maize evapotranspiration over the total growth period under no drought stress.

**Fig 3:**
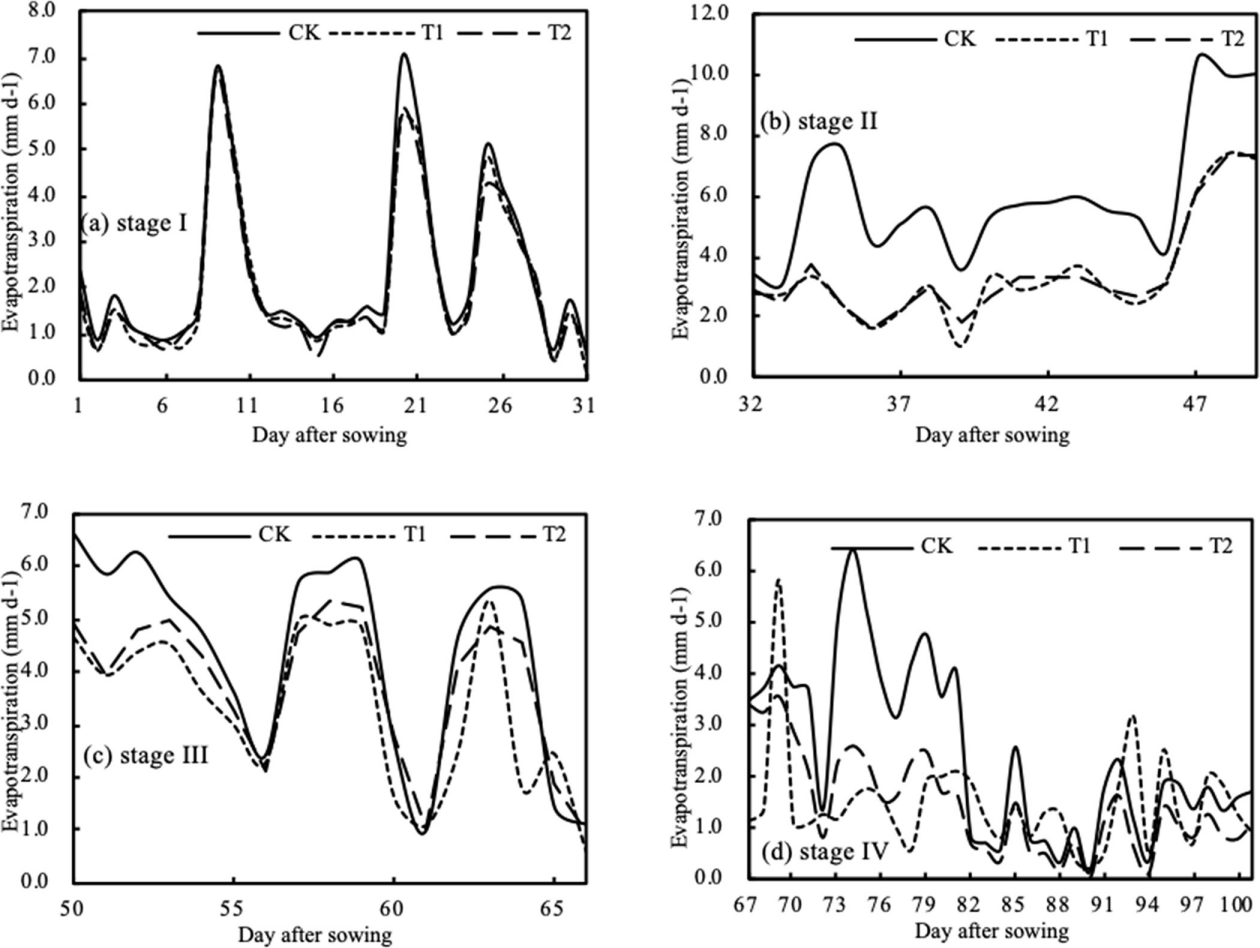
Measured maize evapotranspiration at each growth stage under drought stress.

Based on the actual maize evapotranspiration data under different types of drought stress using lysimeter measurements, we analyzed the change in evapotranspiration over different growth stages (Figs 2 and 3). As shown in the figures, maize evapotranspiration results under different types of drought stress during different growth stages were almost the same. Evapotranspiration at the seedling stage (Stage IV) was minimal, it increased during the elongation stage (Stage II), and reached a high level during the tasseling and silking stage (Stage III), before finally decreasing during the grain filling stage (Stage I).

In Fig 3A, CK, T1, and T2 at the seedling stage represent no drought, slight drought, and medium drought, respectively, and the variability evapotranspiration under the three treatments was almost the same. However, the CK evapotranspiration was a little higher than that of T1 and T2, and average daily evapotranspiration rates for CK, T1, and T2 were 2.318 mm, 2.115 mm, and 2.088 mm, respectively. Differences between the same treatment were minimal and results for slight drought were on average 9.35% lower than the control group. Similarly, in Fig 3B, T1 and T2 at the elongation stage represent slight drought and medium drought, respectively, and there were minimal differences between the different types of treatment. With increasing drought stress duration, however, the differences between T1, T2, and CK in the elongation stage significantly increased, but the difference between slight drought and medium drought during the entire stage was not significant. Evapotranspiration under T1 was 42.71% smaller than that of CK, and T2 was 43.21% smaller than CK. The above analysis indicates that slight drought during the seedling stage did not have a significant influence on the current or later growth stages of maize. Maize evapotranspiration under drought treatment decreased compared to no drought treatment, but the decrease was small. Drought treatment during the elongation stage had a significant influence on maize growth, however; maize evapotranspiration under drought treatment dramatically decreased compared to no drought, which implied that a water deficit reduces maize evapotranspiration, and the greater the water deficit, the greater the decrease in evapotranspiration. Maize was more sensitive to a water deficit during the elongation stage than during the seedling stage.

Fig 3C shows the results for T1 treatment of slight drought during the seedling, elongation, and tasseling and silking stages, and evapotranspiration during the tasseling and silking stage was 24.88% lower than that of CK. T2 involved treatment with slight drought during the seedling stage and medium drought in the elongation and tasseling and silking stages, and evapotranspiration during the tasseling and silking stage was 14.63% lower than that of the control group. Fig 3D shows the results for T1 treatment of slight drought during the seedling, elongation, and tasseling and silking stages, and medium drought in the grain filling stage. Evapotranspiration during the grain filling stage was 38.96% lower than that of CK. T2 involved treatment with slight drought during the seedling stage, medium drought in the elongation and tasseling and silking stages, and severe drought in the grain filling stage. T2 evapotranspiration during the grain filling stage was 41.39% lower than that of CK. The above analysis showed that drought stress during the elongation, tasseling and silking, and grain filling stages significantly reduced maize evapotranspiration. Under continuous drought, the difference in the influence of different types of drought treatments during these stages was small, showing that moderate drought during early growth strengthens maize tolerance to drought conditions.

### Parameter calibration and optimization of angstrom formula

Based on measured total daily solar radiation and sunshine percentage at the Xinmaqiao Experimental Station from 2011 to 2016, we employed the least square method and GA to calibrate and assign the empirical parameters *a* and *b* for the Angstrom formula. To evaluate these two parameters and the suitability of the FAO-recommended parameter, we adopted four statistical indices: mean error, mean absolute error (MAE), root-mean-square error (RMSE), and correlation coefficient [19]. The calibrated parameters and comparisons between the three groups of calculated and observed *R*_*s*_ values are shown in Table 3.

**Table 3:** Comparison of calculated and observed *R*_s_ values based on different *a* and *b*.

Source of Coefficient	Empirical Coefficient	Mean Error σ	MAE (MJ.m^−2^.day^−1^)	RMSE (MJ.m^−2^.dY^−1^)	Calculated Value/Observed Value of *R*_*s*_	Correlation Coefficient *R*
**FAO recommended value**	*a* = 0.250, *b* = 0.500	2.52	5.13	6.68	1.2338	0.8658
**Calibrated value by least square method**	*a* = 0.261, *b* = 0.305	0.71	3.52	4.40	1.0661	0.8694
**Calibrated value by genetic algorithm**	*a* = 0.253, *b* = 0.320	0.66	2.29	4.49	1.0612	0.8706

Table 3 shows that the calculated mean error, MAE, and RMSE values for *R*_*s*_ using the FAO-recommended empirical coefficients *a* and *b*, were all significantly larger than the *R*_*s*_ values calculated using the least square method and genetic algorithm. When the empirical coefficients *a* and *b* were 0.253 and 0.320, respectively, correlation coefficients for the calculated and measured total daily solar radiation values were maximized and the mean error and mean absolute error were minimized. This showed that empirical coefficients *a* = 0.253 and *b =* 0.320 fitted by the genetic algorithm, effectively estimated solar radiation in the Huaibei plain and were better than the least square method results. The data in Table 3 show that solar radiation calculated using the FAO-recommended values was 23.38% higher than the measured value on average.

The calculation of ET_0_ using the Penman-Monteith formula is also influenced by coefficients *a* and *b*. Based on the daily meteorological data, we calculated ET_0_ using the FAO-recommended empirical coefficients *a* = 0.25 and *b* = 0.5, and empirical coefficients *a* = 0.253 and *b =* 0.320. When the FAO-recommended values were used, the average daily ET_0_ from 2011–2016 was 2.48 mm. When the optimized parameters were used, the average daily ET_0_ was 2.07 mm. ET_0_ calculated using the FAO-recommended values was 19.8% higher than the results using optimized parameters.

In summary, the empirical coefficients *a* and *b* calculated using the FAO-recommended values were not suitable for calculating total solar radiation *R*_*s*_ in the Huaibei plain or reference crop evapotranspiration ET_0_. The calculated values were larger than the measured values, which overestimated reference crop evapotranspiration and would be detrimental for water conservation. In contrast, the empirical coefficients *a* and *b* calculated using GA were more suitable for the Huaibei plain.

### Estimated results and analysis of maize evapotranspiration using dual crop coefficient approach and GA

#### Estimated results of maize evapotranspiration under no drought stress

**Fig 4:**
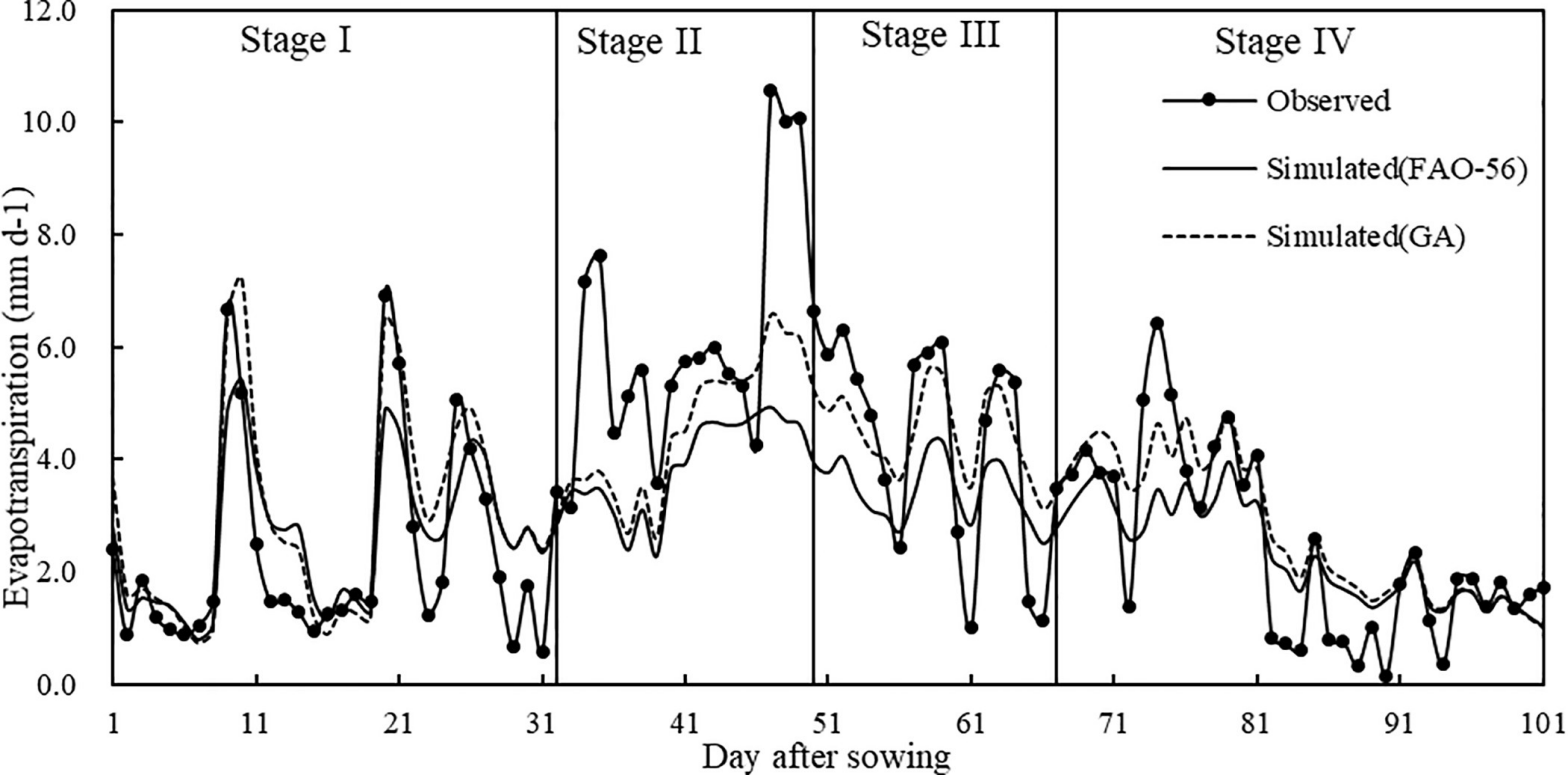
Measured and estimated maize evapotranspiration over the growing period under no drought stress.

Fig 4 shows that maize evapotranspiration during the first half of the seedling stage was small and increased dramatically during the second half of the seedling stage, stayed high during the elongation and tasseling and silking stages and gradually decreased during the grain filling stage. It reached a peak during the later elongation and tasseling and silking stages. Average daily evapotranspiration values during the seedling, elongation, tasseling and silking, and grain filling stages were 2.318 mm, 6.038 mm, 4.398 mm, and 2.441 mm, respectively. The changes in evapotranspiration aligned with actual maize growth. The later seedling, elongation, tasseling and silking, and the earlier grain filling stages were periods of vegetative and reproductive growth when maize has a large demand for water. During the later grain filling stage, maize leaves began to wilt and turn yellow, transpiration decreased significantly, and the daily evapotranspiration continuously declined.

Fig 4 shows that estimated maize evapotranspiration throughout the full growing season using above two methods was basically consistent with the measured results, but the GA results were significantly higher than the FAO-56 results. Combined with the measured and estimated evapotranspiration under no drought stress during each growth stage and across the whole period, as well as the estimation error during each growth stage using FAO-56 as shown in Table 4, RMSE and MAE at each growth stage were higher than the GA results. RMSE, MAE, and MRE over the whole period using GA were 1.33 mm/d, 0.99 mm/d and 1.30%, respectively, or 18.40%, 17.50%, and 91.11% less than the corresponding FAO-56 values. This showed that the GA estimated results were closer to the measured values, and crop coefficients derived using GA should be used to obtain a better fit with the measured value.

**Table 4:** Error results for estimated maize evapotranspiration under no drought stress using the dual crop coefficient approach.

Growth Stage	Measured Value (mm)	Estimated Value (mm)		RMSE (mm/day)		MAE (mm/day)		MRE (*%*)	
		FAO-56	GA	FAO-56	GA	FAO-56	GA	FAO-56	GA
**Stage I**	71.87	81.59	91.21	1.02	1.01	0.82	0.82	13.53	26.90
**Stage II**	108.69	69.17	81.11	2.95	2.26	2.41	1.80	36.36	25.37
**Stage III**	74.76	58.90	76.80	1.60	1.23	1.48	1.02	21.22	2.73
**Stage IV**	85.43	81.22	96.07	1.03	0.93	0.76	0.71	4.92	12.45
**Full growing season**	340.75	290.89	345.19	1.63	1.33	1.20	0.99	14.63	1.30

**Fig 5:**
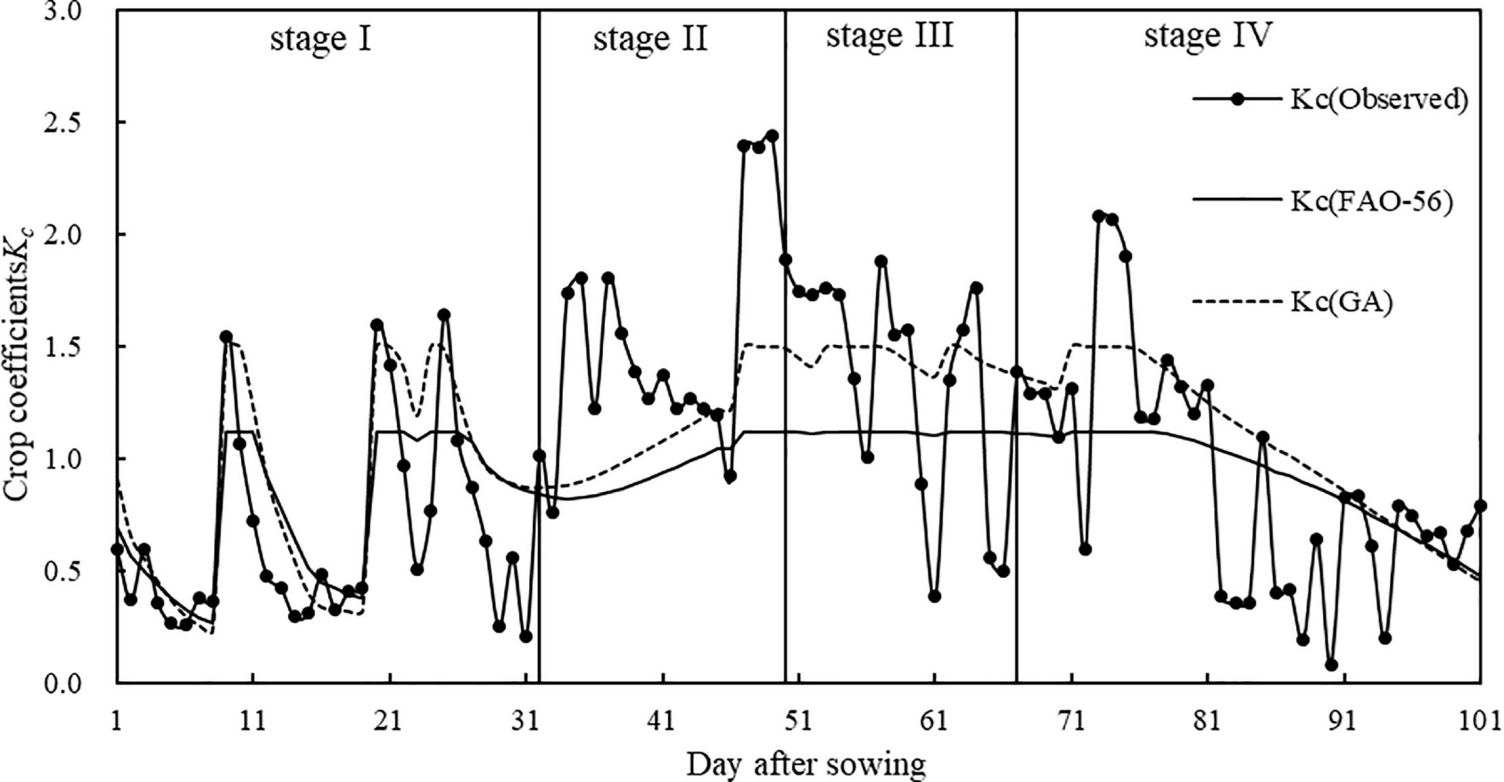
Measured and estimated maize crop coefficients across the full growing season under no drought stress.

We compared the crop coefficients *K*_*cb*ini_, *K*_*cb*mid_, *K*_*cb*end_, and *K*_*c*max_, which are recommended by FAO-56, adjusted by the climatic conditions at the experimental station and optimized by GA (see Section 2.5.1. for the detailed computational process). The crop coefficients using FAO-56 values were 0.150, 1.042, 0.388, and 1.118, and those using GA were 0.155, 1.218, 0.420, and 1.497. Compared with FAO-56, *K*_*cb*ini_, *K*_*cb*mid_, and *K*_*c*max_ obtained by GA significantly increased, while *K*_*cb*end_ significantly decreased. However, the GA crop coefficient *K*_*c*_ obtained using the dual crop coefficient approach was much larger, which was consistent with the change in measured and estimated crop coefficients using the dual crop coefficient approach under no drought stress shown in Fig 5. Therefore, evapotranspiration results from Eq (7) were large. This indicated that the crop coefficients recommended by FAO-56 were lower than those of local maize. In summary, the GA crop coefficients were closer to those of local maize, which led to more accurate estimation of maize evapotranspiration using the dual crop coefficient approach.

#### Estimated results of maize evapotranspiration under drought stress

We adopted the dual crop coefficient approach to estimate maize evapotranspiration under two types of drought stress using the four crop coefficients calibrated by GA, and compared them with the measured results shown in Table 5. The estimated values under the two types of drought stress over the full growing season were higher than the measured values, and results for T1 were more obvious because values during the other stages were all lower, except for the seedling stage. Evapotranspiration over the full growing season was 8.46% lower than the measured value. This showed that the estimation method described in this paper overestimated total maize evapotranspiration. The two sets of RMSE, MAE, and MRE values were used for validation and were 1.149 mm/day, 0.945 mm/day, and 24.792%, and 1.212 mm/day, 1.023 mm/day, and 3.054%, respectively. The estimation results under drought stress were poorer than those obtained under no drought stress, but better than the results using the FAO-56-recommended values. This indicated that GA improved the precision of maize evapotranspiration estimates under drought stress.

**Table 5:** Error results for estimated maize evapotranspiration under drought stress using the dual crop coefficient approach.

Treatment	Growth Stage	Measured Value (mm)	Estimated Value (mm)	RMSE (mm.day^−1^)	MAE (mm.day^−1^)	MRE (%)
**T1**	Stage I	65.58	89.18	1.059	0.853	35.994
	Stage II	61.72	64.32	1.022	0.914	4.207
	Stage III	56.16	55.57	1.001	0.802	−1.055
	Stage IV	52.15	84.95	1.514	1.212	62.905
	Full growing season	235.61	294.02	1.149	0.945	24.792
**T2**	Stage I	64.72	89.18	1.114	0.909	37.801
	Stage II	62.27	41.05	1.391	1.232	−34.075
	Stage III	63.82	55.59	1.155	0.919	−12.893
	Stage IV	50.58	62.93	1.188	1.030	24.423
	Whole growth stage	241.39	248.76	1.212	1.023	3.054

## Conclusions

Slight drought during the seedling stage had a limited influence on maize growth; however, continuous slight drought stress during earlier vegetative growth stages may stimulate the homeostatic mechanism and increase maize tolerance to drought stress.

Slight drought during the seedling stage had minimally reduced maize evapotranspiration in this stage. When drought stress continued after the elongation stage, maize evapotranspiration decreased significantly. Under the same degree of drought, drought stress had a more obvious influence on the middle and later periods of vegetative and reproductive growth.

The values of *a* and *b* obtained by GA calibration improved the ET_0_ accuracy. Because the Xinmaqiao Experimental Station is located in the south end of the Huaibei plain, however, the derived values of *a* and *b* were not applicable to the entire Huaibei plain. More automatic meteorological stations are needed at the irrigation experimental station in the north and central part of the Huaibei plain to increase the observation number and calibration accuracy for total solar radiation.

Based on maize evapotranspiration estimates under no drought stress using the dual crop coefficient approach, we adopted a genetic algorithm to calculate basic crop coefficients *K*_*cb*ini_, *K*_*cb*mid_, *K*_*cb*end_, and their maximum *K*_*c*max_, which were 0.155, 1.218, 0.420, and 1.497, respectively. Next, we applied these crop coefficients and the dual crop coefficient approach to estimate the RMSE, MAE, and MRE for maize evapotranspiration under no drought stress over the full growing season: results were 1.33 mm/day, 0.99 mm/day, and 1.30%, respectively, or 18.40%, 17.50%, and 91.11% lower than the corresponding values calculated using FAO-56-recommended parameters. This indicated that the GA results were closer to the measured values than those obtained using FAO-56, and that estimation using the dual crop coefficient approach based on GA optimization had a better fit with the actual data. Overall maize evapotranspiration accuracy under drought stress during the full growth period was slightly worse than the estimated results under no drought stress, but this analysis showed that the genetic algorithm was able to improve the accurate estimation of maize evapotranspiration under drought stress.

## Supporting information

S1 Data Dataset for this study. (XLSX)
